# Altered Levels of mRNAs for Calcium-Binding/Associated Proteins, Annexin A1, S100A4, and TMEM64, in Peripheral Blood Mononuclear Cells Are Associated with Osteoporosis

**DOI:** 10.1155/2019/3189520

**Published:** 2019-11-11

**Authors:** Ayed A. Dera, Lakshminarayan Ranganath, Roger Barraclough, Sobhan Vinjamuri, Sandra Hamill, Abdullah Y. Mandourah, Dong L. Barraclough

**Affiliations:** ^1^Department of Musculoskeletal Biology, Institute of Ageing and Chronic Disease, University of Liverpool, The William Henry Duncan Building, 6 West Derby Street, Liverpool L7 8TX, UK; ^2^Department of Clinical Biochemistry and Metabolic Medicine, The Royal Liverpool and Broadgreen University Hospital NHS Trust, Prescot Street, Liverpool L7 8XP, UK; ^3^Department of Biochemistry, Institute of Integrative Biology, University of Liverpool, Biosciences Building, Crown Street, Liverpool L69 7ZB, UK; ^4^Department of Nuclear Medicine, The Royal Liverpool and Broadgreen University Hospital NHS Trust, Prescot Street, Liverpool L7 8XP, UK

## Abstract

**Background:**

Osteoporosis is the most common metabolic bone disease in the world. Since osteoporosis is clinically symptomless until the first fracture occurs, early diagnosis is critical. Calcium, along with calcium-binding and calcium-associated proteins, plays an important role in homeostasis, maintaining healthy bone metabolism. This study is aimed at investigating the level of calcium-binding/associated proteins, annexin A1, S100A4, and TMEM64, in peripheral blood mononuclear cells associated with osteoporosis and its clinical significance.

**Methods:**

The levels of mRNAs of annexin A1, S100A4, and TMEM64 in human peripheral blood mononuclear cells were evaluated among 48 osteopenia and 23 osteoporosis patients compared to 17 nonosteoporotic controls. Total RNAs were isolated from clinical samples, and quantitation of mRNA levels was performed using real-time quantitative PCR.

**Results:**

The levels of mRNAs for calcium-binding proteins, annexin A1 and S100A4, and calcium-associated protein, TMEM64, in human peripheral blood mononuclear cells were significantly reduced in osteopenia and osteoporosis patients compared with nonosteoporotic controls (one-way ANOVA, *P* < 0.0001, *P* = 0.039, and *P* = 0.0195, respectively). Annexin A1 and TMEM64 mRNAs were also significantly reduced in female osteoporosis patients over the age of 50 years compared to nonosteoporotic controls (one-way ANOVA, *P* = 0.004 and *P* = 0.0037, respectively). ROC analysis showed that the reduction in the level of mRNA for annexin A1, S100A4, or TMEM64 in the patients' peripheral blood mononuclear cells has a good diagnostic value for osteoporosis.

**Conclusions:**

The results show for the first time that calcium-binding/associated proteins, annexin A1 and TMEM64, could be future diagnostic biomarkers for osteoporosis.

## 1. Introduction

Osteoporosis is an age-related bone disease and has a severe impact on public health and economy worldwide, due to fragility fracture [1]. Since osteoporosis is clinically symptomless until the first fracture, there is a pressing need to identify osteoporosis at an earlier stage. Osteoporosis arises from an imbalance between bone-resorbing osteoclasts and bone-producing osteoblasts [2]. Whilst osteoblasts are derived from bone marrow cells [3], the bone-degrading osteoclasts are derived by cell fusion and differentiation of precursor monocyte blood cells [4], which can be found in the peripheral blood mononuclear cells (PBMCs) of a blood sample [5]. The PBMC fraction could be a source of markers which reflect changes in osteoclast activity in osteoporosis patients and possibly in those with osteopenia, the milder, early form of osteoporosis.

Calcium plays an important role in the development of osteoclasts with the involvement of calcium-binding proteins, such as annexins, S100 proteins, and proteins involved with calcium signaling, such as TMEM64. Three calcium-binding/associated proteins, annexin A1, S100A4, and TMEM64, are expressed in monocyte cells (Gene Expression Atlas: https://www.ebi.ac.uk/gxa/home). The calcium-binding, EF-hand-containing S100 protein, S100A4, has been identified in osteoclasts in developing mouse bone [6]. In mice, knockdown of S100A4 with SiRNA led to a higher bone mass and reduced numbers of osteoclasts [7], suggesting its involvement in some way with bone degradation. Furthermore, it has been suggested that S100A4 protein not only prevents bone excess but also can prevent cortical bone loss in estrogen-deprived mice [8]. Synchronised fusion of mouse osteoclasts has been reported to involve S100A4 and to depend upon a member of the calcium-dependent, phospholipid-binding annexin protein family, annexin A1 [9]. Annexin A1 is functional in the human monocytic cell line, U937 [10], reducing its vascular tethering and transmigration [11]. Furthermore, annexin A1 is an important mediator of the resolution of inflammation [12], in part by enhancing leucocyte apoptosis [13]. Inflammatory diseases have been associated with osteoporotic bone fragility [14].

The transmembrane protein, TMEM64, has been reported to interact with and modulate the activity of the protein, sarcoplasmic endoplasmic reticulum Ca^2+^ ATPase 2 (SERCA2), thereby enhancing RANKL-induced internal calcium oscillations that are a part of the pathway of osteoclast generation [15].

Thus, the aim of this study was to find out whether the levels of mRNAs for annexin A1, S100A4, and TMEM64 were significantly changed in the PBMCs of participants with osteopenia or with osteoporosis compared to nonosteoporotic controls.

## 2. Materials and Methods

### 2.1. Study Subjects

The participants in this study were men and women volunteers living in Merseyside, England, in the area covered by the Royal Liverpool and Broadgreen University Hospital NHS Trust. Participants were recruited from referrals to the Nuclear Medicine Department at the Royal Liverpool University Hospital. Diagnoses of osteopenia and osteoporosis were made in accordance with the WHO guidelines. This study conformed to the principles of the 1964 Helsinki Declaration and its later amendments or comparable ethical standards. The study was carried out under ethical approval from the England Health Research Authority National Research Ethics Service Committee, East of England-Essex (REC reference 15/EE/0051) Ethics Committees. Informed consent was obtained from all participants prior to sample collection. Details of the participants have been described elsewhere [16] (Supplementary [Supplementary-material supplementary-material-1]).

### 2.2. Isolation of Peripheral Blood Mononuclear Cells

Peripheral blood mononuclear cells (PBMCs) were isolated from 25 mL blood samples and stored at -80°C, as described previously [16] including the final 14,000 g centrifugation step to yield a tight pellet of cellular material without maintaining cell viability.

### 2.3. Isolation and Purification of Total RNA

Total RNA from human PBMCs was extracted using a combination of TRIzol reagent and PureLink RNA mini kit (Thermo Fisher, UK), according to the manufacturer's recommendations as described previously [16]. The resulting purified RNAs were eluted in 30 *μ*L of RNase-free water and stored frozen at -80°C until used. RNA concentrations and purity were measured using a Thermo Scientific NanoDrop™ 2000 spectrophotometer.

### 2.4. Quantitation of mRNA Levels Using Real-Time Quantitative PCR (RT-qPCR)

Reverse transcription reactions were carried out using the RT-First Strand kit, RT-qPCR Primer Assays (Qiagen, UK), according to the manufacturer's recommendations and as described previously [16] on 500 ng isolated RNA in a total volume of 20 *μ*L with a no reverse transcriptase, no DNA controls. Reverse transcription reactions were diluted 1 : 20, and 10 *μ*L samples were subjected to RT-qPCR for annexin A1, S100A4, TMEM64, and glyceraldehyde-3-phosphate dehydrogenase (GPDH) cDNAs, using the primers indicated in Table 1, with a SYBR Green PCR kit (Qiagen, UK) in a Roche LightCycler 96 Real-Time PCR system (Roche, UK). Each set of reactions was accompanied by the no-reverse-transcription, no-DNA control sample. The relative levels of each RNA were determined from the Ct value after normalization with control mRNA, GPDH using the 2^-*ΔΔ*CT^ method [17]. Ct values > 35 obtained from RT-qPCR were considered to be below the level of detection of the methodology.

### 2.5. Statistical Analyses

Statistical analysis between two groups used Student's *t*-test and between multiple groups used ANOVA with post hoc Bonferroni correction. Diagnostic values were determined using receiver operator characteristic curves. Statistical analyses were carried out using Stats Direct 3 (Altrincham, Cheshire). *P* values of <0.05 were considered significant as described previously [18].

## 3. Results

### 3.1. The Levels of mRNAs of Calcium-Binding/Associated Proteins, Annexin A1, S100A4, and TMEM64, in the Peripheral Blood Mononuclear Cells Are Reduced in Osteoporosis Patients

**Figure 1:**
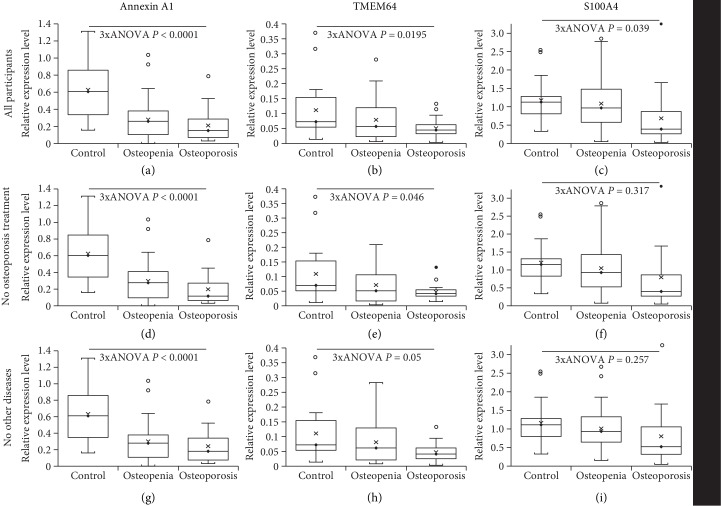
Relative levels of mRNAs in peripheral blood mononuclear cells associated with osteoporosis. Levels of mRNAs encoding annexin A1 (a, d, g), TMEM64 (b, e, h), and S100A4 (c, f, i) proteins were determined in the peripheral blood mononuclear cell preparations of nonosteoporotic control subjects and patients suffering from osteopenia or osteoporosis shown as box and whisker plots. The levels are shown for all participants (a, b, c), for participants who were not receiving treatment for osteoporosis (d, e, f), and for participants who were not suffering from other disorders (g, h, i). On each box and whisker plot, the black diamond shows the median value, the cross shows the mean value, and white and black circles denote outliers of 1.5 times and 3 times the interquartile range, respectively.

The relative levels of mRNAs of calcium-binding/associated proteins, annexin A1, S100A4, and TMEM64, in isolated PBMC preparations were each significantly different between nonosteoporosis control, osteopenia, or osteoporosis groups of participants (one-way ANOVA, annexin A1 mRNA, Figure 1(a), *F*(2 : 85) = 16.7, *P* < 0.0001; TMEM64 mRNA, Figure 1(b), *F*(2 : 84) = 4.13, *P* = 0.0195; S100A4, Figure 1(c), *F*(2 : 84) = 3.4, *P* = 0.039). For annexin A1 mRNA, the strongly significant decline was evident between nonosteoporotic and osteopenia groups (Figure 1(a) and Table 2; nonosteoporotic control group vs. osteopenia, 95% CI 0.208 to 0.476, *P* < 0.0001; nonosteoporotic control group vs. osteoporosis, 95% CI 0.262 to 0.566, *P* < 0.0001; osteoporosis vs. osteopenia, 95% CI -0.192 to 0.049, *P* = 0.242, post hoc Bonferroni correction). For TMEM64, there was a gradual reduction in the order: nonosteoporotic control, osteopenia, and osteoporosis groups (Figure 1(b) and Table 2; nonosteoporotic control group vs. osteopenia, 95% CI -0.004 to 0.0729, *P* = 0.0797; nonosteoporotic control group vs. osteoporosis, 95% CI 0.019 to 0.106, *P* = 0.0052; osteoporosis vs. osteopenia, 95% CI -0.062 to 0.006, *P* = 0.1, post hoc Bonferroni correction). For S100A4 mRNA, a significant reduction was evident between osteopenia and osteoporosis groups (Figure 1(c) and Table 2; nonosteoporotic control group vs. osteopenia, 95% CI -0.302 to 0.47, *P* = 0.666; nonosteoporotic control group vs. osteoporosis, 95% CI 0.053 to 0.925, *P* = 0.0286; osteoporosis vs. osteopenia, 95% CI -0.751 to -0.057, *P* = 0.0229, post hoc Bonferroni correction).

### 3.2. Analysis of Levels of mRNAs for Calcium-Binding/Associated Proteins Associated with Treatment of Osteoporosis and Other Disorders

For annexin A1 or TMEM64, the significant associations of reduced levels of mRNA with osteopenia and osteoporosis were not affected when participants receiving treatment for osteoporosis were excluded from the analysis (annexin A1 mRNA, one-way ANOVA, *F*(2, 68) = 12.7, *P* < 0.0001; Figure 1(d) and Table 3; nonosteoporotic control group vs. osteopenia, 95% CI 0.18 to 0.478, *P* < 0.0001; nonosteoporotic control group vs. osteoporosis, 95% CI 0.232 to 0.603, *P* < 0.0001; osteoporosis vs. osteopenia, 95% CI -0.248 to 0.071, *P* = 0.272; TMEM64 mRNA, one-way ANOVA, *F*(2, 67) = 3.2, *P* = 0.046; Figure 1(e) and Table 3; nonosteoporotic control group vs. osteopenia, 95% CI -0.00096 to 0.0784, *P* = 0.056; nonosteoporotic control group vs. osteoporosis, 95% CI 0.011 to 0.109, *P* = 0.0168; osteoporosis vs. osteopenia, 95% CI -0.063 to 0.02, *P* = 0.305; Bonferroni post hoc test). When participants with nonosteoporotic medical disorders were excluded, for annexin A1 or TMEM64, the significant associations of reduced levels of mRNA with osteopenia and osteoporosis were not affected (annexin A1 mRNA, one-way ANOVA, *F*(2, 66) = 11.8, *P* < 0.0001; Figure 1(g) and Table 4; nonosteoporotic control group vs. osteopenia, 95% CI 0.176 to 0.477, *P* < 0.0001; nonosteoporotic control group vs. osteoporosis, 95% CI 0.202 to 0.557, *P* < 0.0001; osteoporosis vs. osteopenia, 95% CI -0.206 to 0.1, *P* = 0.491; TMEM mRNA, one-way ANOVA, *F*(2, 65) = 3.1, *P* = 0.05; Figure 1(h) and Table 4; nonosteoporotic control group vs. osteopenia, 95% CI -0.013 to 0.075, *P* = 0.166; nonosteoporotic control group vs. osteoporosis, 95% CI 0.013 to 0.117, *P* = 0.016; osteoporosis vs. osteopenia, 95% CI -0.078 to 0.01, *P* = 0.132, Bonferroni post hoc test).

However, for S100A4, the significant association between the reduction in S100A4 mRNA and osteoporosis was lost when participants either receiving treatment for osteoporosis or participants with nonosteoporotic medical disorders were excluded from the analysis (osteoporosis treatment removed, one-way ANOVA, *F*(2, 67) = 1.17, *P* = 0.317; Figure 1(f) and Table 3; other diseases removed one-way ANOVA, *F*(2, 66) = 1.39, *P* = 0.257; Figure 1(i) and Table 4), showing that the relationship between S100A4 mRNA level and osteoporosis was potentially affected by treatment for osteoporosis and by the presence of other disorders, whereas the relationship between annexin A1 or TMEM64 mRNA and osteoporosis was not.

### 3.3. The Levels of mRNAs of Calcium-Binding/Associated Proteins Are Reduced in the Peripheral Blood Mononuclear Cells of Female Osteoporosis Patients Aged over 50 Years

**Figure 2:**
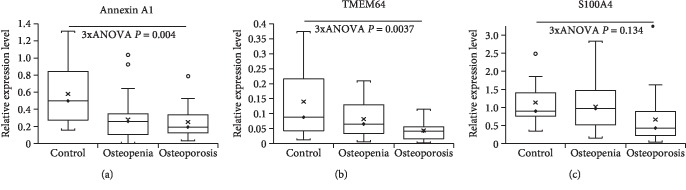
Relative levels of mRNAs in peripheral blood mononuclear cells associated with osteoporosis in females over the age of 50. Levels of mRNAs encoding annexin A1 (a), TMEM64 (b), and S100A4 (c) proteins were determined in peripheral blood mononuclear cell preparations of nonosteoporotic control subjects and patients suffering from osteopenia or osteoporosis. On each box and whisker plot, the black diamond shows the median value, the cross shows the mean value, and white and black circles denote outliers of 1.5 times and 3 times the interquartile range, respectively.

To find out whether the same results were obtained in female osteoporosis patients aged over 50 years specifically, the analyses were confined to the 63 female subjects over 50 years of age. All three mRNAs showed the same trend of a decrease in the mRNA level in the order: nonosteoporotic control, osteopenia, and osteoporosis observed when all patients were included. Annexin A1 and TMEM64 mRNAs showed a significant reduction between nonosteoporotic, osteopenia, and osteoporosis participants (annexin A1 mRNA, one-way ANOVA, *F*(2, 60) = 5.95, *P* = 0.004; Figure 2(a) and Table 5; nonosteoporotic controls vs. osteopenia, 95% CI 0.114 to 0.493, *P* = 0.0021; nonosteoporotic control group vs. osteoporosis, 95% CI 0.125 to 0.539, *P* = 0.0022; osteoporosis vs. osteopenia, 95% CI -0.175 to 0.118, *P* = 0.699, Bonferroni post hoc test; TMEM64 mRNA, one-way ANOVA, *F*(2, 60) = 6.14, *P* = 0.0037; Figure 2(b) and Table 5; nonosteoporotic control group vs. osteopenia, 95% CI 0.008 to 0.108, *P* = 0.0245; nonosteoporotic control group vs. osteoporosis, 95% CI 0.041 to 0.15, *P* = 0.0009; osteoporosis vs. osteopenia, 95% CI -0.077 to 0.0009, *P* = 0.056, Bonferroni post hoc test). However, the changes in S100A4 mRNA between the nonosteoporotic control group, osteopenia, or osteoporosis patients were not significant in the female participants aged over 50 years (one-way ANOVA, *F*(2, 59) = 2.08, *P* = 0.134; Figure 2(c) and Table 5).

### 3.4. The Levels of mRNAs for Annexin A1, TMEM64, or S100A4 in Peripheral Blood Mononuclear Cells from Participants with or without Chronic Inflammatory Disorders

Osteoporosis has been associated with chronic inflammatory disorders. In order to find out whether the reduction in the levels of mRNAs for annexin A1, TMEM64, or S100A4 in peripheral blood mononuclear cells was associated with the presence of chronic diseases, the participants with osteopenia or osteoporosis were divided into those without and those with reported chronic diseases or undergoing treatment with steroids. The levels of annexin A1, TMEM64, or S100A4 mRNAs in PBMCs were not significantly different between those with and those without known chronic inflammatory disorders (Supplementary [Supplementary-material supplementary-material-1]).

### 3.5. Diagnostic Value of Calcium-Binding/Associated Protein mRNAs for Osteoporosis

**Figure 3:**
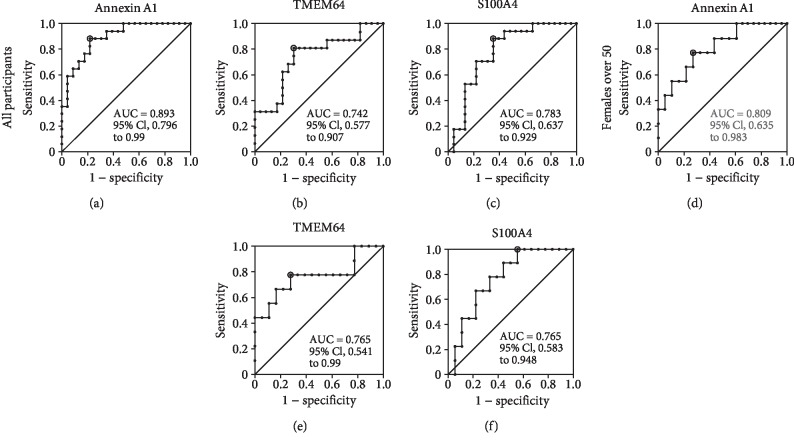
Diagnostic values of annexin A1, TMEM64, and S100A4 mRNAs for nonosteoporotic controls vs osteoporosis. ROC curves between nonosteoporotic controls and osteoporosis sufferers are shown for the mRNAs of annexin A1 (a, d), TMEM64 (b, e), and S100A4 (c, f) for all participants (a, b, c) and female participants over the age of 50 (d, e, f). The circled point indicates the optimum cut-off.

**Figure 4:**
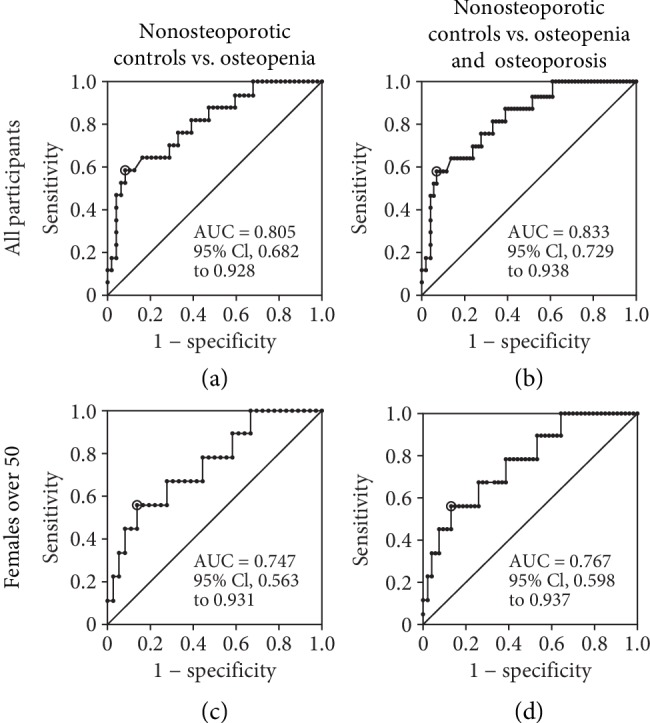
Diagnostic values of annexin A1 for nonosteoporotic controls vs osteopenia and osteoporosis. ROC curves are shown for the mRNA for annexin A1 for all participants (a, b) and female participants over the age of 50 (c, d), comparing nonosteoporotic controls with osteopenia (a, c) and nonosteoporotic controls with osteopenia and osteoporosis (b, d). The circled point indicates the optimum cut-off.

The levels of mRNAs for annexin A1, S100A4, and TMEM64 were reduced in PBMCs from osteoporosis patients compared to nonosteoporotic controls; thus, the presence of these mRNAs marked the nonosteoporotic condition. ROC curve analysis for nonosteoporotic controls and osteoporosis showed that, when all participants were included, the areas under the ROC curves for annexin A1, TMEM64, and S100A4 were 0.893, (Figure 3(a), 95% CI 0.796 to 0.99; sensitivity 0.882, specificity 0.783), 0.742 (Figure 3(b), 95% CI 0.577 to 0.907; sensitivity 0.813, specificity 0.696), and 0.783 (Figure 3(c), 95% CI 0.637 to 0.929; sensitivity 0.882, specificity 0.652), respectively. The reduction of neither TMEM64 nor S100A4 discriminated well nonosteoporotic controls from osteopenia (area under the ROC curves, 0.6 and 0.55, respectively, not shown) or osteopenia and osteoporosis combined (area under the ROC curves, 0.646 and 0.627, respectively, not shown). However, for annexin A1, when nonosteoporotic controls were compared with osteopenia, the area under the ROC curve was 0.805 (Figure 4(a), 95% CI 0.682 to 0.928; sensitivity 0.588, specificity 0.917) and when compared with osteopenia and osteoporosis combined, the area under the ROC curve was 0.833 (Figure 4(b), 95% CI 0.729 to 0.938; sensitivity 0.588, specificity 0.93).

When the analysis was confined to females over the age of 50 years, similar results were obtained, with areas under the ROC curves for annexin A1, TMEM64, and S100A4 mRNAs being 0.809 (Figure 3(d), 95% CI 0.635 to 0.983; sensitivity 0.778, specificity 0.722), 0.765 (Figure 3(e), 95% CI 0.541 to 0.99; sensitivity 0.778, specificity 0.722), and 0.765 (Figure 3(f), 95% CI 0.583 to 0.948; sensitivity 1.0, specificity 0.444), respectively. Neither TMEM64 nor S100A4 mRNAs discriminated well nonosteoporotic controls from osteopenia (area under the ROC curves, 0.614 and 0.543, respectively, not shown) or osteopenia and osteoporosis combined (area under the ROC curves, 0.667 and 0.618, respectively, not shown). However, for annexin A1 mRNA, when nonosteoporotic controls were compared with osteopenia, the area under the curve was 0.747 (Figure 4(c), 95% CI 0.563 to 0.931; sensitivity 0.556, specificity 0.861) and when compared with osteopenia and osteoporosis combined, the area under the curve was 0.767 (Figure 4(d), 95% CI 0.598 to 0.937; sensitivity 0.556, specificity 0.87).

## 4. Discussion

Three mRNAs, encoding calcium-binding/associated proteins, ANXA1, S100A4, and TMEM64, were found to be at a lower level in the PBMC preparations from osteoporosis patients than nonosteoporotic controls. The reductions of S100A4 mRNA levels were found to be due to participants with other disorders, those receiving existing treatments for osteoporosis, males, and under 50-year olds. In contrast, the observed reductions in annexin A1 or TMEM64 mRNAs were not affected by any of these factors.

Annexin A1 protein and, to a lesser extent, TMEM64 protein are found in the CD14+/CD16-negative monocytes [19] (Gene Expression Atlas: https://www.ebi.ac.uk/gxa/home). These cells have been shown to be the likely source of osteoclasts by differentiation *in vitro* of cells from normal human subjects [20]. For TMEM64 mRNA in PBMCs, the observed reduction in the level with osteoporosis in the present experiments seems unexpected in view of previous results with TMEM64 knockout mice, in which reduced levels of TMEM64 mRNA resulted in a phenotype of increased bone mass [21]. Apart from possible species differences between mouse and human, such as observed for annexin A1 [9], the difference between the present results and the TMEM64 mouse knockout results could arise from the cell preparations being examined in the two studies. In the knockout mice, there was a reduction in bone-associated, fully differentiated osteoclasts [21], whereas in the present study, the TMEM64 mRNA levels were examined in the blood PBMC fraction, which contains monocytes as yet undifferentiated into osteoclasts [4].

Annexin A1 has been reported to be present in the human U937 cultured monocyte cell line, where it has been reported to reduce adhesion of U937 cells to bone marrow endothelial cells by directly interacting with *α*4 integrin and competing with binding of the endothelial *α*4 integrin receptor, VCAM-1 [11]. A reduction in annexin A1 mRNA, and thus possibly a reduction in annexin A1 protein, in monocytes could therefore result in increased retention of monocytes by endothelial cells in the bone marrow and reduced numbers in peripheral blood mononuclear cells.

However, the PBMC fractions consist of only 20-40% monocytes and a low percentage of polymorphonuclear cells (1-4%), but a majority (60-80%) of cells in PBMC preparations are lymphocytes [22]. TMEM64 mRNA is present at a low level in both T and B lymphocytes and is more abundant in natural killer cells (Gene Expression Atlas: https://www.ebi.ac.uk/gxa/home). Annexin A1 (lipocortin 1) has been reported to be present at a consistent level in many T lymphocyte subtypes and at a higher level in natural killer cells [19], but not present in B lymphocytes (Gene Expression Atlas: https://www.ebi.ac.uk/gxa/home). Thus, it is possible that the observed reductions in annexin A1 and TMEM64 mRNAs arise in the lymphocyte populations in the PBMC preparations. It is not known whether there are specific differences in the cell types populating PBMCs of nonosteoporotic individuals and osteopenia and osteoporosis patients. However, T lymphocytes [23, 24] and natural killer cells [25] have been reported to contribute to osteoporosis by their activation and production of osteoclastogenic cytokines, but the observation that various T cell subtypes can exhibit either osteoclastogenic, e.g., Thy 17, or antiosteoclastogenic activity, e.g., Thy1 [26], suggests that there is a balance between pro- and antiosteoclastic activity in T cell subtypes. Whilst, little, if anything, is known about any possible role of TMEM64 in these processes in T lymphocytes, the presence of annexin A1, on the other hand, has been widely associated with the resolution of inflammatory disorders [12, 13, 27]. Thus, a change in the balance of T cell osteoclastogenic activity, arising from the reduced annexin A1 mRNA levels in PBMCs from osteopenia/osteoporosis patients, could contribute to their osteopenia/osteoporosis. However, this might not be consistent with the observation on the present group of participants that the levels of annexin mRNA in the PBMCs of participants with chronic inflammatory disorders were not significantly different from those without chronic inflammatory disorders, either when all participants or when only the female-over-50 group were considered.

ROC curve analysis for the changes in these mRNAs showed moderate accuracy for the areas under the curves [28]. Annexin A1 exhibited the best trade-off of sensitivity against specificity of the three possible markers for all participants when nonosteoporotic controls were compared with osteoporosis (area under the curve, 0.893), with osteopenia (area under the curve, 0.805), or with osteopenia and osteoporosis combined (area under the curve, 0.833) or when only females over the age of 50 were included (areas under the curves, 0.809, 0.747, and 0.767, respectively).

In conclusion, this study demonstrates for the first time that the levels of mRNAs of annexin A1 and TMEM64 in peripheral blood mononuclear cells are reduced in association with osteoporosis, at least in this group of patients with loss of annexin A1 mRNA in peripheral blood mononuclear cells being a good marker for osteopenia/osteoporosis.

## Figures and Tables

**Table 1:** Qiagen primers used to amplify specific mRNAs by RT-qPCR.

mRNA	Gene symbol	Accession number	Primer sequence	Qiagen catalogue
Annexin A1	ANXA1	NM_000700.2	5′TGTTTTAGCTCTGCTAAAAACTCCAG 3′	PPH02882E
S100 calcium-binding protein A4	S100A4	NM_002961	5′CAACTTGGACAGCAACAGGGAC 3′	PPH01313E
Transmembrane protein 64	TMEM64	NM_001008495.3	5′TTGGACTGCTTCCTACCCAGCTTC 3′	PPH10400A
Glyceraldehyde-3-phosphate dehydrogenase	GAPDH	NM_001256799	5′GGCGCTGCCAAGGCTGTGGGCA 3′	PPH00150F

**Table 2:** Summary of statistical analysis on all participants.

mRNA	Compared sample groups (*n* = number of participants)	95% confidence interval	*P* value (Bonferroni correction)
Annexin A1	Nonosteoporotic control (*n* = 17) vs. osteopenia (*n* = 48)	0.208 to 0.476	<0.0001
	Nonosteoporotic control (*n* = 17) vs. osteoporosis (*n* = 23)	0.262 to 0.566	<0.0001
	Osteoporosis (*n* = 23) vs. osteopenia (*n* = 48)	-0.192 to 0.049	0.242

TMEM64	Nonosteoporotic control (*n* = 16) vs. osteopenia (*n* = 48)	-0.004 to 0.0729	0.0797
	Nonosteoporotic control (*n* = 16) vs. osteoporosis (*n* = 23)	0.019 to 0.106	0.0052
	Osteoporosis (*n* = 23) vs. osteopenia (*n* = 48)	-0.062 to 0.006	0.1

S100A4	Nonosteoporotic control (*n* = 17) vs. osteopenia (*n* = 47)	-0.302 to 0.47	0.666
	Nonosteoporotic control (*n* = 17) vs. osteoporosis (*n* = 23)	0.053 to 0.925	0.0286
	Osteoporosis (*n* = 23) vs. osteopenia (*n* = 47)	-0.751 to -0.057	0.0229

**Table 3:** Summary of statistical analysis on participants with no osteoporosis treatment.

mRNA	Compared sample groups (*n* = number of participants)	95% confidence interval	*P* value (Bonferroni correction)
Annexin A1	Nonosteoporotic control (*n* = 17) vs. osteopenia (*n* = 40)	0.18 to 0.478	<0.0001
	Nonosteoporotic control (*n* = 17) vs. osteoporosis (*n* = 14)	0.232 to 0.603	<0.0001
	Osteoporosis (*n* = 14) vs. osteopenia (*n* = 40)	-0.248 to 0.071	0.272

TMEM64	Nonosteoporotic control (*n* = 16) vs. osteopenia (*n* = 40)	-0.00096 to 0.0784	0.056
	Nonosteoporotic control (*n* = 16) vs. osteoporosis (*n* = 14)	0.011 to 0.109	0.0168
	Osteoporosis (*n* = 14) vs. osteopenia (*n* = 40)	-0.063 to 0.02	0.305

S100A4	Nonosteoporotic control (*n* = 17) vs. osteopenia (*n* = 39)	-0.273 to 0.556	0.498
	Nonosteoporotic control (*n* = 17) vs. osteoporosis (*n* = 14)	-0.124 to 0.906	0.134
	Osteoporosis (*n* = 14) vs. osteopenia (*n* = 39)	-0.694 to 0.195	0.267

**Table 4:** Summary of statistical analysis on participants without nonosteoporotic medical disorders.

mRNA	Compared sample groups (*n* = number of participants)	95% confidence interval	*P* value (Bonferroni correction)
Annexin A1	Nonosteoporotic control (*n* = 17) vs. osteopenia (*n* = 36)	0.176 to 0.477	<0.0001
	Nonosteoporotic control (*n* = 17) vs. osteoporosis (*n* = 16)	0.202 to 0.557	<0.0001
	Osteoporosis (*n* = 16) vs osteopenia (*n* = 36)	-0.206 to 0.1	0.491


TMEM64	Nonosteoporotic control (*n* = 16) vs. osteopenia (*n* = 36)	-0.013 to 0.075	0.166
	Nonosteoporotic control (*n* = 16) vs. osteoporosis (*n* = 16)	0.013 to 0.117	0.016
	Osteoporosis (*n* = 16) vs osteopenia (*n* = 36)	-0.078 to 0.01	0.132

S100A4	Nonosteoporotic control (*n* = 17) vs. osteopenia (*n* = 36)	-0.224 to 0.54	0.413
	Nonosteoporotic control (*n* = 17) vs. osteoporosis (*n* = 16)	-0.077 to 0.828	0.102
	Osteoporosis (*n* = 16) vs. osteopenia (*n* = 36)	-0.608 to 0.172	0.269

**Table 5:** Summary of statistical analysis on female participants over the age of 50.

mRNA	Compared sample groups (*n* = number of participants)	95% confidence interval	*P* value (Bonferroni correction)
Annexin A1	Nonosteoporotic control (*n* = 9) vs. osteopenia (*n* = 36)	0.114 to 0.493	0.0021
	Nonosteoporotic control (*n* = 9) vs. osteoporosis (*n* = 18)	0.125 to 0.539	0.0022
	Osteoporosis (*n* = 18) vs. osteopenia (*n* = 36)	-0.175 to 0.118	0.699

TMEM64	Nonosteoporotic control (*n* = 9) vs. osteopenia (*n* = 36)	0.008 to 0.108	0.0245
	Nonosteoporotic control (*n* = 9) vs. osteoporosis (*n* = 18)	0.041 to 0.15	0.0009
	Osteoporosis (*n* = 18) vs. osteopenia (*n* = 36)	-0.077 to 0.0009	0.056

S100A4	Nonosteoporotic control (*n* = 9) vs. osteopenia (*n* = 35)	-0.406 to 0.601	0.7
	Nonosteoporotic control (*n* = 9) vs. osteoporosis (*n* = 18)	-0.095 to 1.01	0.103
	Osteoporosis (*n* = 18) vs. osteopenia (*n* = 35)	-0.7486 to 0.033	0.072

## Data Availability

The data used to support the findings of this study are available from the corresponding author upon request.

## Supplementary Materials

Supplementary Materials Supplementary Figure S1: relative levels of mRNAs for annexin A1, TMEM64, and S100A4 were determined in the peripheral blood mononuclear cells of osteopenia and osteoporosis participants. Box and whisker plots show the levels of mRNAs in participants with or without chronic inflammatory disorders/treatment with steroids. Supplementary Table S1: summary of characteristics of clinical samples.
