# Supplementary material for: Altered Levels of mRNAs for Calcium-Binding/Associated Proteins, Annexin A1, S100A4, and TMEM64, in Peripheral Blood Mononuclear Cells Are Associated with Osteoporosis
Source: Dis Markers. 2019 Nov 11;2019:3189520. doi: 10.1155/2019/3189520 (PMC6877971; doi:10.1155/2019/3189520)
Supplement: Supplementary Materials — Supplementary Figure S1: relative levels of mRNAs for annexin A1, TMEM64, and S100A4 were determined in the peripheral blood mononuclear cells of osteopenia and osteoporosis participants. Box and whisker plots show the levels of mRNAs in participants with or without chronic inflammatory disorders/treatment with steroids. Supplementary Table S1: summary of characteristics of clinical samples. [file 3189520.f1.pdf]

Altered levels of mRNAs for calcium binding / associated proteins, Annexin A1, S100A4 and TMEM64 in peripheral blood mononuclear cells are associated with osteoporosis

Ayed A. Dera[1,5], Lakshminarayan Ranganath[2], Roger Barraclough[3], Sobhan Vinjamuri[4], Sandra Hamill[4], Abdullah Y. Mandourah[1,6] & Dong L. Barraclough[1*]

Supplementary Figure S1. Relative levels of mRNAs for annexin A1, TMEM64 and S100A4 were determined in the peripheral blood mononuclear cells of osteopenia and osteoporosis participants. Box and whisker plots show the levels of mRNAs in participants with or without chronic inflammatory disorders/treatment with steroids. *P* values, Student's t test.

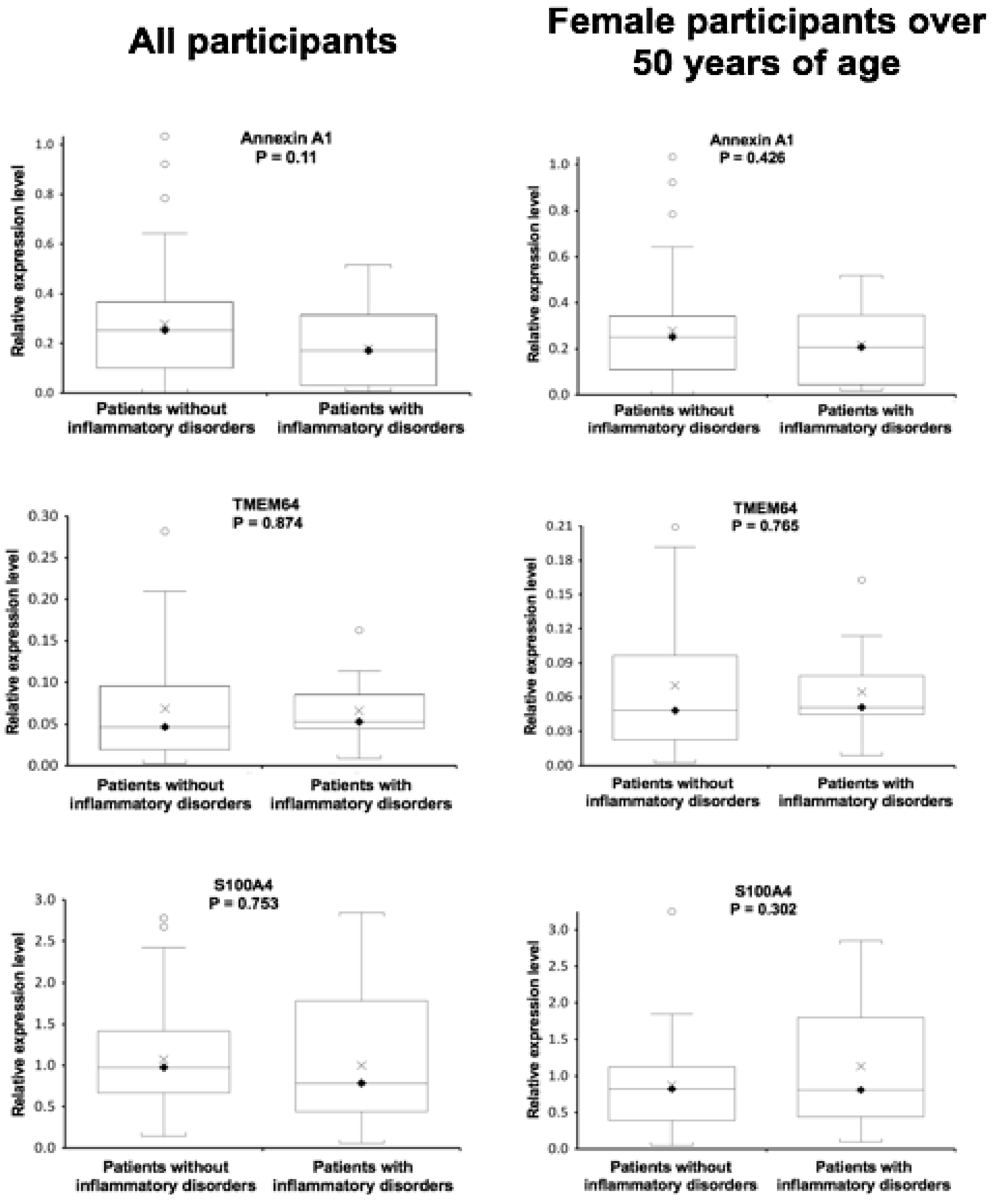

Supplementary Table S1. Summary of characteristics of clinical samples*.

Clinical category	Non-osteoporotic control	Osteopenia	Osteoporosis
Number of participants	17 (14 females / 3 males)	48 (38 females / 10 males)	23 (19 females / 4 males)
Participants' age (mean ± SD years)	56 ± 18.9	65 ± 9.7	69 ± 13.2
Number of participants > 50 years of age	10	45	22
Age of participants > 50 years of age (mean ± SD years)	70 ± 5.5	67 ± 7.8	71 ± 8.2
Number of patients with other diseases (chronic inflammatory diseases)**	0	12 (11)	7 (4)
Number of patients treated with Bisphosphonate or Denosumab ***	0	8	9
Number of participants with fracture history	4	24	9
BMD (g/cm^2) (Mean± SD)	0.964 ± 0.108	0.841 ± 0.107	0.710 ± 0.074
Lumbar 2-4 *T*-score (Mean± SD) ****	0.427	-1	-2.604
Femoral neck *T*-score (Mean± SD)	-0.18	-1.285	-2.296

* Characteristics of these patients have been described previously (Dera AA, Ranganath L, Barraclough R, Vinjamuri S, Hamill S, Barraclough DL (2019) Cathepsin Z as a novel potential biomarker for osteoporosis. Sci Rep 9:9752, |https://doi.org/10.1038/s41598-019-46068-0).

** Twelve osteopenia patients (nine female and three male) and seven osteoporosis patients (five female and two male) had other diseases including hypertension and type 2 diabetes and chronic inflammatory diseases: arthritis, asthma, coeliac disease, COPD, Crohn's disease, osteoarthritis, rheumatoid arthritis or were being treated with steroids.

*** Eight osteopenia (five female and three male) and seven female osteoporosis patients were being treated with bisphosphonate, such as Alendronate, Ibandronate, Risedronate and Zoledronic acid. Two female osteoporosis patients were treated with Denosumab.

**** Patients were categorised as osteopenia or osteoporosis based on either a low femoral neck or lumbar 2-4 *T*-score, thus some osteopenia patients with low femoral neck scores had normal lumbar 2-4 *T* scores.
